# The genome sequence of the Brindled Beauty, *Lycia hirtaria *(Clerck, 1759)

**DOI:** 10.12688/wellcomeopenres.19650.1

**Published:** 2023-07-12

**Authors:** Douglas Boyes, Peter W.H. Holland, University of Oxford and Wytham Woods Genome Acquisition Lab, Darwin Tree of Life Barcoding collective, Wellcome Sanger Institute Tree of Life programme, Wellcome Sanger Institute Scientific Operations: DNA Pipelines collective, Tree of Life Core Informatics collective, Darwin Tree of Life Consortium

**Affiliations:** 1 UK Centre for Ecology & Hydrology, Wallingford, England, UK; 2 University of Oxford, Oxford, England, UK

**Keywords:** Lycia hirtaria, brindled beauty, genome sequence, chromosomal, Lepidoptera

## Abstract

We present a genome assembly from an individual male *Lycia hirtaria* (the Brindled Beauty; Arthropoda; Insecta; Lepidoptera; Geometridae). The genome sequence is 552.0 megabases in span. Most of the assembly is scaffolded into 14 chromosomal pseudomolecules, including the Z sex chromosome. The mitochondrial genome has also been assembled and is 15.58 kilobases in length.

## Species taxonomy

Eukaryota; Metazoa; Eumetazoa; Bilateria; Protostomia; Ecdysozoa; Panarthropoda; Arthropoda; Mandibulata; Pancrustacea; Hexapoda; Insecta; Dicondylia; Pterygota; Neoptera; Endopterygota; Amphiesmenoptera; Lepidoptera; Glossata; Neolepidoptera; Heteroneura; Ditrysia; Obtectomera; Geometroidea; Geometridae; Ennominae; *Lycia*; *Lycia hirtaria* (Clerck, 1759) (NCBI:txid326963).

## Background

Several moths in the subfamily Ennominae, family Geometridae, have winged males and flightless females, with wing reduction probably having evolved multiple times ( Wahlberg *et al.*, 2010). To understand the genetic basis and the selective pressures underpinning this trait, species with flightless females must be compared to close relatives with winged females. The Brindled Beauty *Lycia hirtaria* is an example of this group in which both males and females are fully winged: in each sex the forewings are smoky-grey with black cross-lines. Interspecific crosses have been made between *L. hirtaria* and several related species, although the offspring are usually infertile ( Ford, 1967; Harrison, 1916). In crosses between *L. hirtaria* and species with wingless females, partially winged hybrids are sometimes obtained. When the male is *L. hirtaria*, some of these crosses also give sex ratio distortion with a predominance of phenotypic males ( Harrison, 1916; Harrison, 1919; Harrison & Doncaster, 1914). In the case of infertile offspring produced by crossing *L. hirtaria* with *L. zonaria*, examination of hybrids suggests that major karyotype differences cause disruption of chromosome paring during meiosis in the F _1_ generation ( Harrison & Doncaster, 1914).

*L. hirtaria* is found in woodland and suburban areas across northern Europe and further east through Russia to Japan ( GBIF Secretariat, 2022; Wagner, 2023). In Britain and Ireland, the moth is widespread but not usually common, and is recorded most frequently in the southeast of England ( Randle *et al.*, 2019). The adult moth is on the wing in early spring, peaking in April in southern England, with larvae feeding in summer on the leaves of deciduous trees including *Prunus*, *Crataegus* and *Salix*; the pupal stage overwinters. Abundance of the species in Britain has declined by over 70% since 1970 ( Randle *et al.*, 2019); in the 19th century it was sufficiently abundant in London to cause widespread defoliation of trees ( Newman, 1869).

The complete genome of *Lycia hirtaria* was determined as part of the Darwin Tree of Life project. The assembled genome will contribute to the growing set of resources for studying insect ecology and evolution.

## Genome sequence report

**Figure 1.**
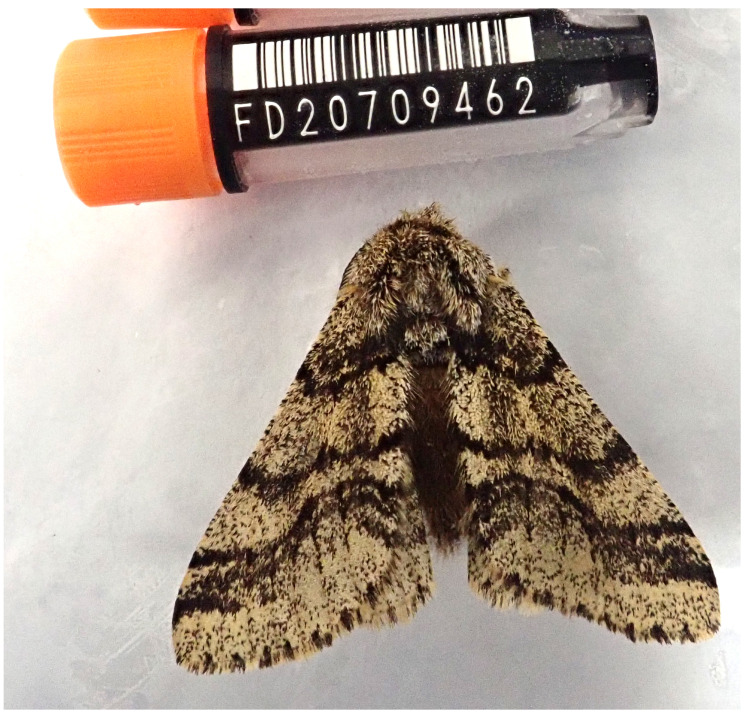
Photograph of the *Lycia hirtaria* (ilLycHirt1) specimen used for genome sequencing.

The genome was sequenced from one male *Lycia hirtaria* ( Figure 1) collected from Wytham Woods, Oxfordshire, UK (51.77, –1.34). A total of 40-fold coverage in Pacific Biosciences single-molecule HiFi long reads was generated. Primary assembly contigs were scaffolded with chromosome conformation Hi-C data. Manual assembly curation corrected three missing joins or mis-joins and removed one haplotypic duplication, reducing the scaffold number by 4.76%.

**Figure 2.**
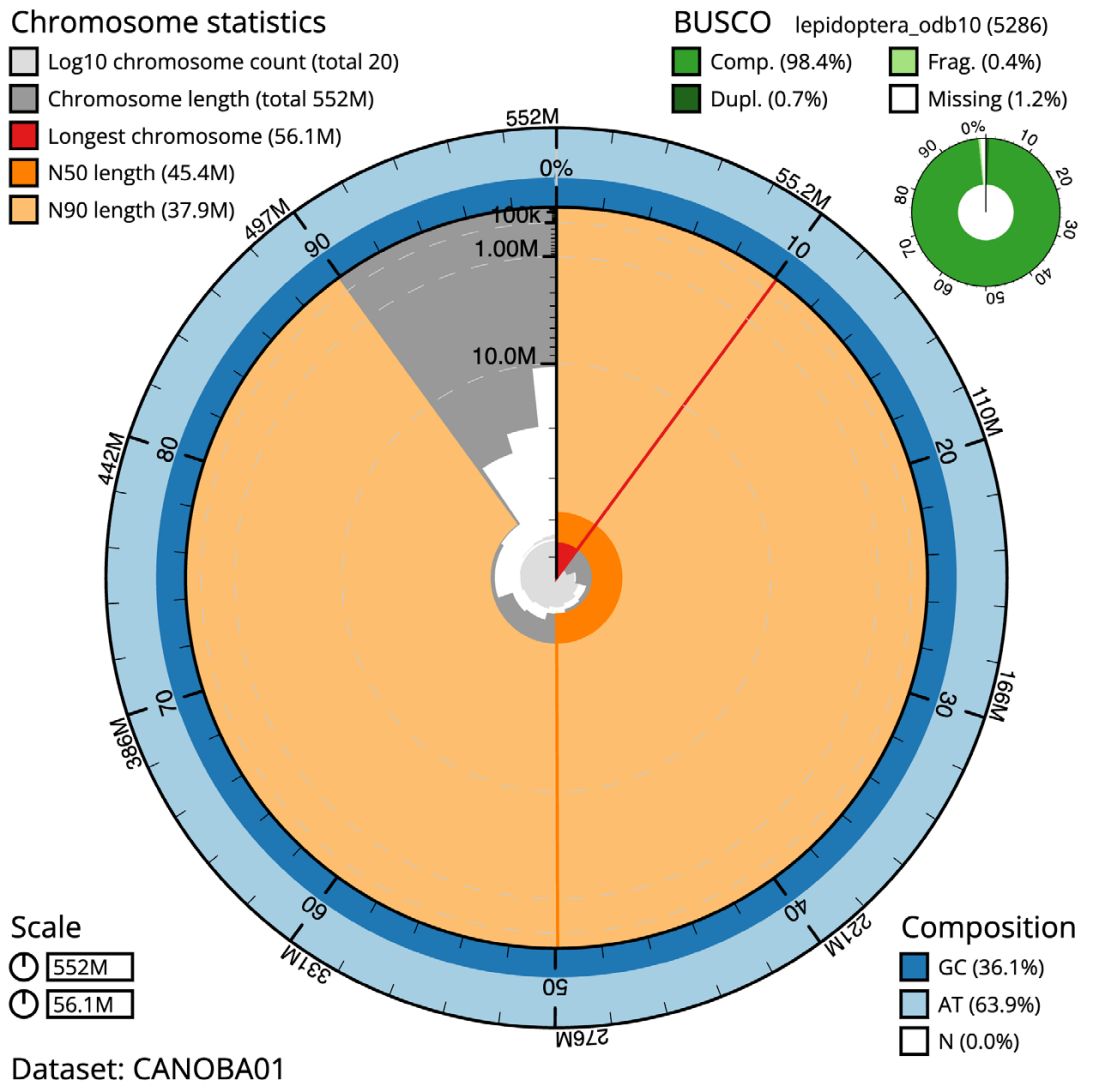
Genome assembly of *Lycia hirtaria*, ilLycHirt1.1: metrics. The BlobToolKit Snailplot shows N50 metrics and BUSCO gene completeness. The main plot is divided into 1,000 size-ordered bins around the circumference with each bin representing 0.1% of the 551,971,097 bp assembly. The distribution of scaffold lengths is shown in dark grey with the plot radius scaled to the longest scaffold present in the assembly (56,061,216 bp, shown in red). Orange and pale-orange arcs show the N50 and N90 scaffold lengths (45,432,280 and 37,909,223 bp), respectively. The pale grey spiral shows the cumulative scaffold count on a log scale with white scale lines showing successive orders of magnitude. The blue and pale-blue area around the outside of the plot shows the distribution of GC, AT and N percentages in the same bins as the inner plot. A summary of complete, fragmented, duplicated and missing BUSCO genes in the lepidoptera_odb10 set is shown in the top right. An interactive version of this figure is available at https://blobtoolkit.genomehubs.org/view/Lycia%20hirtaria/dataset/CANOBA01/snail.

The final assembly has a total length of 552.0 Mb in 19 sequence scaffolds with a scaffold N50 of 45.4 Mb ( Table 1). Most (99.92%) of the assembly sequence was assigned to 14 chromosomal-level scaffolds, representing 13 autosomes and the Z sex chromosome. Chromosome-scale scaffolds confirmed by the Hi-C data are named in order of size ( Figure 2– Figure 5; Table 2). While not fully phased, the assembly deposited is of one haplotype. Contigs corresponding to the second haplotype have also been deposited. The mitochondrial genome was also assembled and can be found as a contig within the multifasta file of the genome submission.

**Table 1.** Genome data for *Lycia hirtaria*, ilLycHirt1.1.

Project accession data		
Assembly identifier	ilLycHirt1.1	
Species	*Lycia hirtaria*	
Specimen	ilLycHirt1	
NCBI taxonomy ID	326963	
BioProject	PRJEB56733	
BioSample ID	SAMEA10107033	
Isolate information	ilLycHirt1, male: thorax (DNA sequencing), head (Hi-C scaffolding)	
Assembly metrics *		*Benchmark*
Consensus quality (QV)	67.1	*≥ 50*
*k*-mer completeness	100%	*≥ 95%*
BUSCO **	C:98.4%[S:97.7%,D:0.7%], F:0.4%,M:1.2%,n:5,286	*C ≥ 95%*
Percentage of assembly mapped to chromosomes	99.92%	*≥ 95%*
Sex chromosomes	Z chromosome	*localised homologous pairs*
Organelles	Mitochondrial genome assembled	*complete single alleles*
Raw data accessions		
PacificBiosciences SEQUEL II	ERR10395969	
Hi-C Illumina	ERR10378040	
Genome assembly		
Assembly accession	GCA_947563715.1	
*Accession of alternate haplotype*	GCA_947563705.1	
Span (Mb)	552.0	
Number of contigs	83	
Contig N50 length (Mb)	11.8	
Number of scaffolds	19	
Scaffold N50 length (Mb)	45.4	
Longest scaffold (Mb)	56.1	

* Assembly metric benchmarks are adapted from column VGP-2020 of “Table 1: Proposed standards and metrics for defining genome assembly quality” from ( Rhie *et al.*, 2021). ** BUSCO scores based on the lepidoptera_odb10 BUSCO set using v5.3.2. C = complete [S = single copy, D = duplicated], F = fragmented, M = missing, n = number of orthologues in comparison. A full set of BUSCO scores is available at https://blobtoolkit.genomehubs.org/view/Lycia%20hirtaria/dataset/CANOBA01/busco.

**Figure 3.**
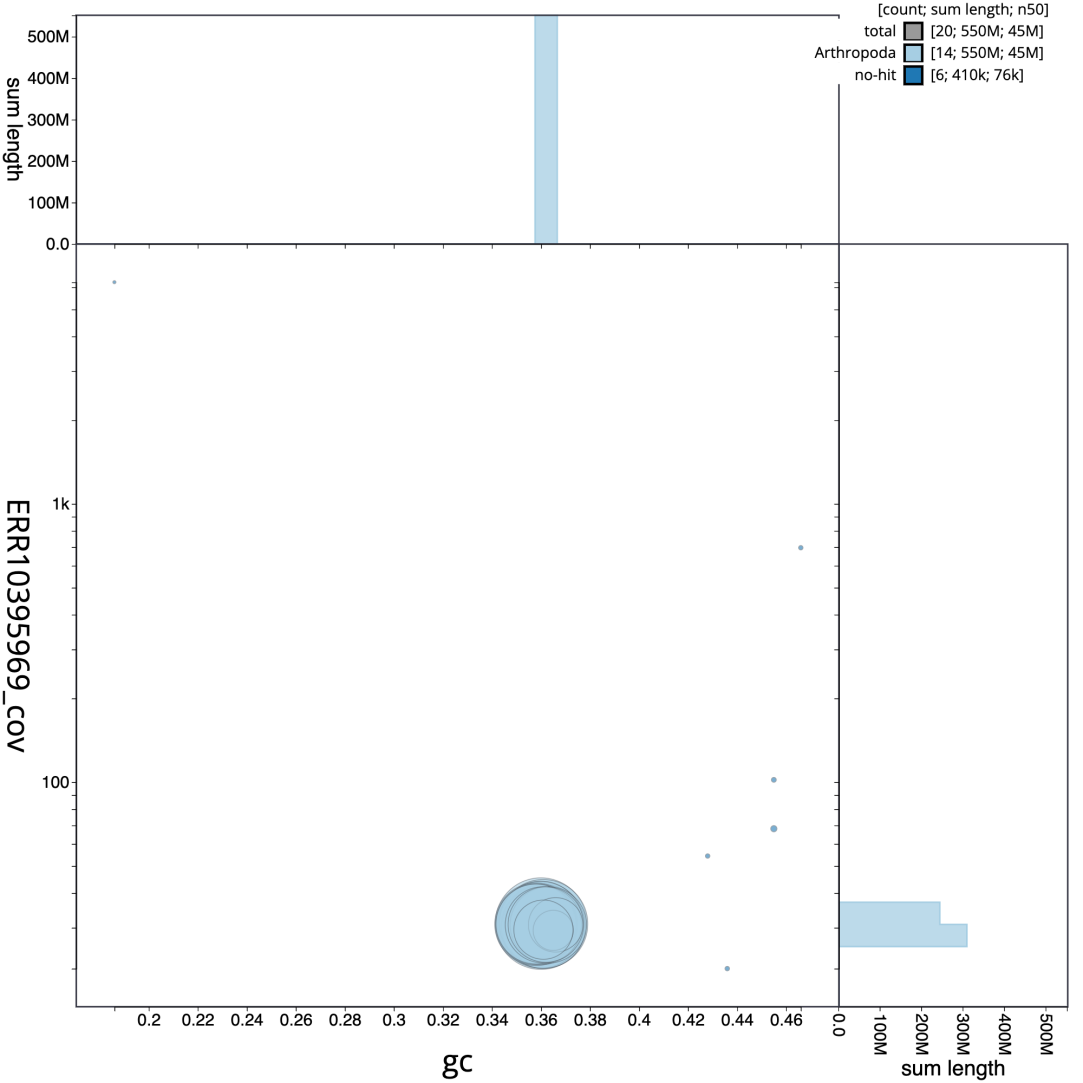
Genome assembly of *Lycia hirtaria*, ilLycHirt1.1: BlobToolKit GC-coverage plot. Scaffolds are coloured by phylum. Circles are sized in proportion to scaffold length. Histograms show the distribution of scaffold length sum along each axis. An interactive version of this figure is available at https://blobtoolkit.genomehubs.org/view/Lycia%20hirtaria/dataset/CANOBA01/blob.

**Figure 4.**
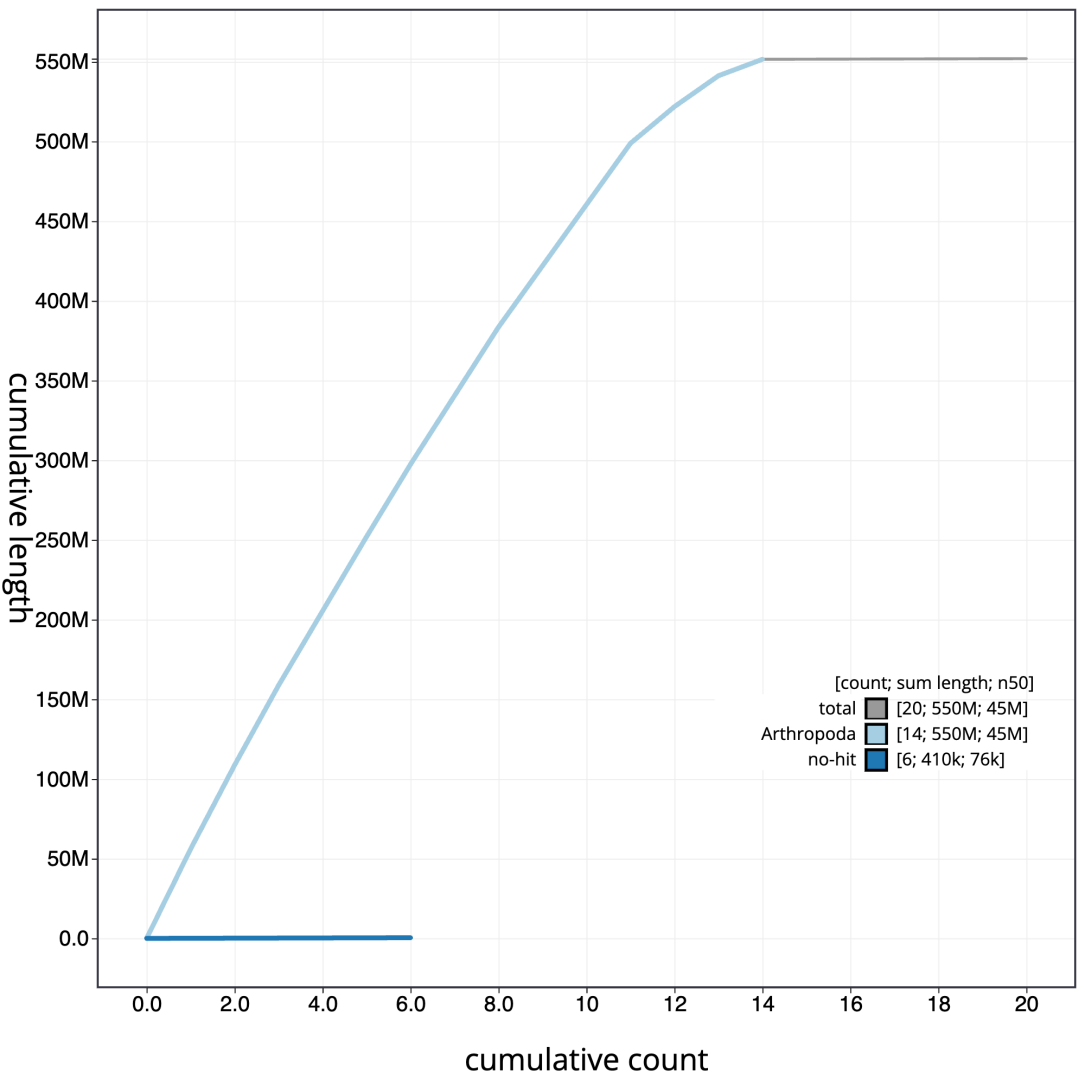
Genome assembly of *Lycia hirtaria*, ilLycHirt1.1: BlobToolKit cumulative sequence plot. The grey line shows cumulative length for all scaffolds. Coloured lines show cumulative lengths of scaffolds assigned to each phylum using the buscogenes taxrule. An interactive version of this figure is available at https://blobtoolkit.genomehubs.org/view/Lycia%20hirtaria/dataset/CANOBA01/cumulative.

**Figure 5.**
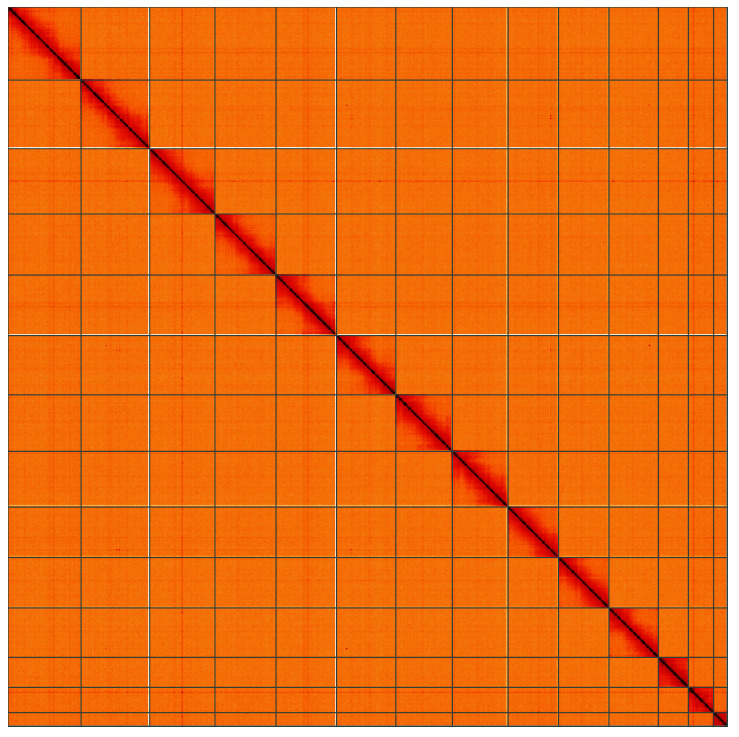
Genome assembly of *Lycia hirtaria*, ilLycHirt1.1: Hi-C contact map of the ilLycHirt1.1 assembly, visualised using HiGlass. Chromosomes are shown in order of size from left to right and top to bottom. An interactive version of this figure may be viewed at https://genome-note-higlass.tol.sanger.ac.uk/l/?d=eaCUaTdDS5yUWAKuI097OA.

**Table 2.** Chromosomal pseudomolecules in the genome assembly of *Lycia hirtaria*, ilLycHirt1.

INSDC accession	Chromosome	Length (Mb)	GC%
OX387375.1	1	56.06	36.0
OX387376.1	2	52.63	36.0
OX387377.1	3	50.05	36.0
OX387378.1	4	46.87	36.0
OX387379.1	5	46.41	36.0
OX387380.1	6	45.43	36.0
OX387381.1	7	43.42	36.0
OX387382.1	8	42.67	36.0
OX387383.1	9	38.71	36.5
OX387384.1	10	38.67	36.0
OX387385.1	11	37.91	36.0
OX387387.1	12	19.38	36.5
OX387388.1	13	10.32	36.5
OX387386.1	Z	23.03	36.0
OX387389.1	MT	0.02	18.5

The estimated Quality Value (QV) of the final assembly is 67.1 with *k*-mer completeness of 100%, and the assembly has a BUSCO v5.3.2 completeness of 98.4% (single = 97.7%, duplicated = 0.7%), using the lepidoptera_odb10 reference set ( *n* = 5,286).

Metadata for specimens, spectral estimates, sequencing runs, contaminants and pre-curation assembly statistics can be found at https://links.tol.sanger.ac.uk/species/326963.

## Methods

### Sample acquisition and nucleic acid extraction

A male *Lycia hirtaria* (specimen ID Ox001108, individual ilLycHirt1) was collected using a light trap in Wytham Woods, Oxfordshire (biological vice-county Berkshire), UK (latitude 51.77, longitude –1.34) on 2021-03-31. Douglas Boyes (University of Oxford) collected and identified the specimen. The specimen was snap-frozen on dry ice.

DNA was extracted at the Tree of Life laboratory, Wellcome Sanger Institute (WSI). The ilLycHirt1 sample was weighed and dissected on dry ice with tissue set aside for Hi-C sequencing. Thorax tissue was cryogenically disrupted to a fine powder using a Covaris cryoPREP Automated Dry Pulveriser, receiving multiple impacts. High molecular weight (HMW) DNA was extracted using the Qiagen MagAttract HMW DNA extraction kit. HMW DNA was sheared into an average fragment size of 12–20 kb in a Megaruptor 3 system with speed setting 30. Sheared DNA was purified by solid-phase reversible immobilisation using AMPure PB beads with a 1.8X ratio of beads to sample to remove the shorter fragments and concentrate the DNA sample. The concentration of the sheared and purified DNA was assessed using a Nanodrop spectrophotometer and Qubit Fluorometer and Qubit dsDNA High Sensitivity Assay kit. Fragment size distribution was evaluated by running the sample on the FemtoPulse system.

### Sequencing

Pacific Biosciences HiFi circular consensus DNA sequencing libraries were constructed according to the manufacturers’ instructions. DNA sequencing was performed by the Scientific Operations core at the WSI on a Pacific Biosciences SEQUEL II (HiFi) instrument. Hi-C data were also generated from head tissue of ilLycHirt1 using the Arima2 kit and sequenced on the Illumina NovaSeq 6000 instrument.

### Genome assembly, curation and evaluation

Assembly was carried out with Hifiasm ( Cheng *et al.*, 2021) and haplotypic duplication was identified and removed with purge_dups ( Guan *et al.*, 2020). The assembly was then scaffolded with Hi-C data ( Rao *et al.*, 2014) using YaHS ( Zhou *et al.*, 2023). The assembly was checked for contamination and corrected as described previously ( Howe *et al.*, 2021). Manual curation was performed using HiGlass ( Kerpedjiev *et al.*, 2018) and Pretext ( Harry, 2022). The mitochondrial genome was assembled using MitoHiFi ( Uliano-Silva *et al.*, 2022), which runs MitoFinder ( Allio *et al.*, 2020) or MITOS ( Bernt *et al.*, 2013) and uses these annotations to select the final mitochondrial contig and to ensure the general quality of the sequence.

A Hi-C map for the final assembly was produced using bwa-mem2 ( Vasimuddin *et al.*, 2019) in the Cooler file format ( Abdennur & Mirny, 2020). To assess the assembly metrics, the *k*-mer completeness and QV consensus quality values were calculated in Merqury ( Rhie *et al.*, 2020). This work was done using Nextflow ( Di Tommaso *et al.*, 2017) DSL2 pipelines “sanger-tol/readmapping” ( Surana *et al.*, 2023a) and “sanger-tol/genomenote” ( Surana *et al.*, 2023b). The genome was analysed within the BlobToolKit environment ( Challis *et al.*, 2020) and BUSCO scores ( Manni *et al.*, 2021; Simão *et al.*, 2015) were calculated.

Table 3 contains a list of relevant software tool versions and sources.

**Table 3.** Software tools: versions and sources.

Software tool	Version	Source
BlobToolKit	4.1.5	https://github.com/blobtoolkit/blobtoolkit
BUSCO	5.3.2	https://gitlab.com/ezlab/busco
Hifiasm	0.16.1-r375	https://github.com/chhylp123/hifiasm
HiGlass	1.11.6	https://github.com/higlass/higlass
Merqury	MerquryFK	https://github.com/thegenemyers/MERQURY.FK
MitoHiFi	2	https://github.com/marcelauliano/MitoHiFi
PretextView	0.2	https://github.com/wtsi-hpag/PretextView
purge_dups	1.2.3	https://github.com/dfguan/purge_dups
sanger-tol/genomenote	v1.0	https://github.com/sanger-tol/genomenote
sanger-tol/readmapping	1.1.0	https://github.com/sanger-tol/readmapping/tree/1.1.0
YaHS	yahs-1.1.91eebc2	https://github.com/c-zhou/yahs

### Wellcome Sanger Institute – Legal and Governance

The materials that have contributed to this genome note have been supplied by a Darwin Tree of Life Partner. The submission of materials by a Darwin Tree of Life Partner is subject to the **‘Darwin Tree of Life Project Sampling Code of Practice’**, which can be found in full on the Darwin Tree of Life website here. By agreeing with and signing up to the Sampling Code of Practice, the Darwin Tree of Life Partner agrees they will meet the legal and ethical requirements and standards set out within this document in respect of all samples acquired for, and supplied to, the Darwin Tree of Life Project. 

Further, the Wellcome Sanger Institute employs a process whereby due diligence is carried out proportionate to the nature of the materials themselves, and the circumstances under which they have been/are to be collected and provided for use. The purpose of this is to address and mitigate any potential legal and/or ethical implications of receipt and use of the materials as part of the research project, and to ensure that in doing so we align with best practice wherever possible. The overarching areas of consideration are:

- Ethical review of provenance and sourcing of the material
- Legality of collection, transfer and use (national and international)

Each transfer of samples is further undertaken according to a Research Collaboration Agreement or Material Transfer Agreement entered into by the Darwin Tree of Life Partner, Genome Research Limited (operating as the Wellcome Sanger Institute), and in some circumstances other Darwin Tree of Life collaborators.

## Data Availability

European Nucleotide Archive: *Lycia hirtaria* (brindled beauty). Accession number PRJEB56733; https://identifiers.org/ena.embl/PRJEB56733. ( Wellcome Sanger Institute, 2022) The genome sequence is released openly for reuse. The *Lycia hirtaria* genome sequencing initiative is part of the Darwin Tree of Life (DToL) project. All raw sequence data and the assembly have been deposited in INSDC databases. The genome will be annotated using available RNA-Seq data and presented through the Ensembl pipeline at the European Bioinformatics Institute. Raw data and assembly accession identifiers are reported in Table 1.

## Author information

Members of the University of Oxford and Wytham Woods Genome Acquisition Lab are listed here: https://doi.org/10.5281/zenodo.4789928.

Members of the Darwin Tree of Life Barcoding collective are listed here: https://doi.org/10.5281/zenodo.4893703.

Members of the Wellcome Sanger Institute Tree of Life programme are listed here: https://doi.org/10.5281/zenodo.4783585.

Members of Wellcome Sanger Institute Scientific Operations: DNA Pipelines collective are listed here: https://doi.org/10.5281/zenodo.4790455.

Members of the Tree of Life Core Informatics collective are listed here: https://doi.org/10.5281/zenodo.5013541.

Members of the Darwin Tree of Life Consortium are listed here: https://doi.org/10.5281/zenodo.4783558.
