# Supplementary material for: The genome sequence of the Brindled Beauty, Lycia hirtaria (Clerck, 1759)
Source: Wellcome Open Res. 2023 Jul 12;8:303. [Version 1] doi: 10.12688/wellcomeopenres.19650.1 (PMC11255552; doi:10.12688/wellcomeopenres.19650.1)
Supplement: Peer review history

# Reviewer report: Reviewer response for version 1

**DOI:** 10.21956/wellcomeopenres.21767.r87427

**Published:** 2024-07-17

**Authors:** Roberto Feuda

**Affiliations:** 1 University of Leicester, Leicester, England, UK

In this article, Boyes and Holland present the genome sequence of a male *Lycia hirtaria.* The rationale for sequencing this genome is well described, as its comparison will inform the genomic basis of wing gain and retention and the overall ecology of this declining species. The entire process, from DNA extraction to genome assembly, is documented. Additionally, the genome and the raw reads are available.

Are sufficient details of methods and materials provided to allow replication by others?

Yes

Is the rationale for creating the dataset(s) clearly described?

Yes

Are the datasets clearly presented in a useable and accessible format?

Yes

Are the protocols appropriate and is the work technically sound?

Yes

Reviewer Expertise:

Genomics and Evolutionary biology

I confirm that I have read this submission and believe that I have an appropriate level of expertise to confirm that it is of an acceptable scientific standard.

# Reviewer report: Reviewer response for version 1

**DOI:** 10.21956/wellcomeopenres.21767.r87425

**Published:** 2024-07-17

**Authors:** Ludvik M Gomulski

**Affiliations:** 1 University of Pavia, Pavia, Italy

The manuscript describes the nuclear and mitochondrial genome sequences obtained from a single male of the Brindled beauty moth, *Lycia hirtaria* from Wytham Woods, Oxfordshire. One of the reasons for sequencing this species is to understand the genetic basis and selective pressures underlying the presence of flightless females in several species within the subfamily Ennominae, to which *L. hirtaria* pertains, and whose females are fully winged. The genome sequences appear to be of very high quality and the methods and analyses used are well described and appropriate, and the datasets are readily accessible.

Are sufficient details of methods and materials provided to allow replication by others?

Yes

Is the rationale for creating the dataset(s) clearly described?

Yes

Are the datasets clearly presented in a useable and accessible format?

Yes

Are the protocols appropriate and is the work technically sound?

Yes

Reviewer Expertise:

Behaviour, physiology and functional genetics of insects, especially mosquitoes

I confirm that I have read this submission and believe that I have an appropriate level of expertise to confirm that it is of an acceptable scientific standard.

# Reviewer report: Reviewer response for version 1

**DOI:** 10.21956/wellcomeopenres.21767.r84129

**Published:** 2024-05-27

**Authors:** James B. Whitfield

**Affiliations:** 1 University of Illinois at Urbana-Champaign, Champaign, IL, USA

This manuscript provides the critical data details from the recently acquired sequence of the genome of the Brindled Beauty, *Lycia hirtaria* (Clerck), a common and notably polymorphic species of geometrid moth which is of additional interest in having both sexes fully winged while close relatives have flightless females. The new genome can thus serve as a useful comparison when studying the genes responsible for the winged/wingless shift. Some genetic experiments concerning this shift are mentioned, but it is not clear whether additional genomes of close relatives have been or are currently being sequenced. It would have been useful to know what is the closest relative for which the genomes sequence is known, and whether the chromosomal organization of this genome is unusual or not. 

 The assembly appears to be of suitably high quality to be useful for such comparisons, and is based on the sequence from a single male. The paper as a whole, and the methods in particular, are very clearly written and cleanly illustrated. The methodology and results reflect the current state of the art.

Are sufficient details of methods and materials provided to allow replication by others?

Yes

Is the rationale for creating the dataset(s) clearly described?

Yes

Are the datasets clearly presented in a useable and accessible format?

Yes

Are the protocols appropriate and is the work technically sound?

Yes

Reviewer Expertise:

Systematics and ecology of parasitoids of Lepidoptera; comparative genomics of parasitoid wasps and their viruses

I confirm that I have read this submission and believe that I have an appropriate level of expertise to confirm that it is of an acceptable scientific standard.
